# Supplementary material for: The interactome of intact mitochondria by cross-linking mass spectrometry provides evidence for coexisting respiratory supercomplexes
Source: Mol Cell Proteomics. 2017 Dec 8;17(2):216–32. doi: 10.1074/mcp.RA117.000470 (PMC5795388; doi:10.1074/mcp.RA117.000470)
Supplement: Supplemental Data [file supp_RA117.000470_133922_0_supp_23978_fzffwf.zip › spectra_annotation/mito_spectra_annotation/76-1-105-1-9-1.pdf]

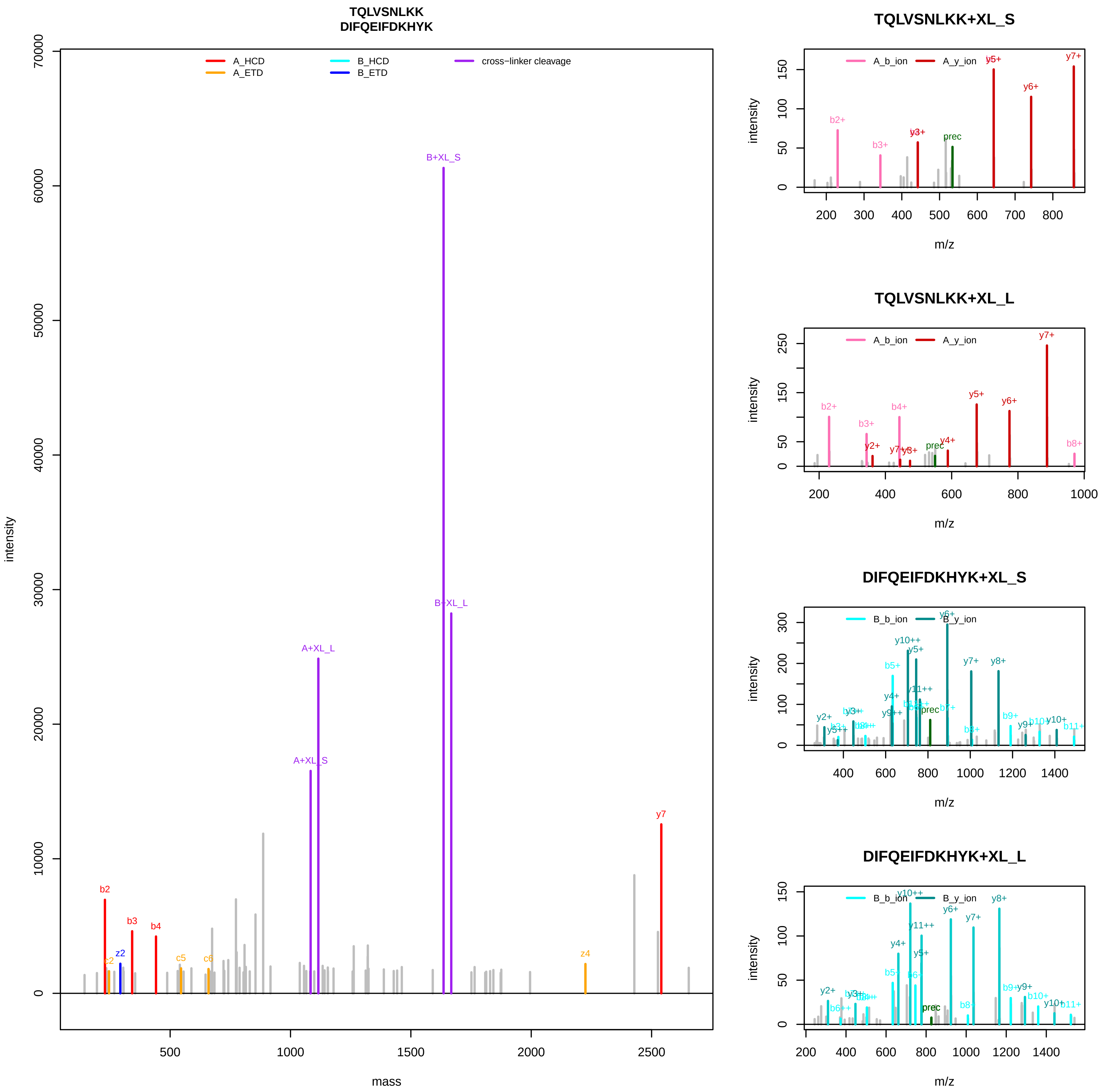
TQLVSNLKK
DIFQEIFDKHYK
A_HCD
A_ETD
B_HCD
B_ETD
cross−linker cleavage
B+XL_S
B+XL_L
A+XL_L
A+XL_S
b2
b3
b4
c2
z2
c5
c6
z4
y7
intensity
mass
TQLVSNLKK+XL_S
A_b_ion
A_y_ion
b2+
b3+
y3+
prec
y5+
y6+
y7+
m/z
TQLVSNLKK+XL_L
b4+
y2+
y4+
b8+
DIFQEIFDKHYK+XL_S
B_b_ion
B_y_ion
y6+
y10++
b5+
y11++
y8+
b9+
b10+
y10+
b11+
DIFQEIFDKHYK+XL_L
